# Building a brain watch: Crafting Accurate and High-Precision Personalized Plans for Optimal Brain Performance and Sustained Functionality

**DOI:** 10.1016/j.jarlif.2025.100013

**Published:** 2025-04-18

**Authors:** Ara S. Khachaturian

**Affiliations:** a Brain Watch Coalition of the Campaign to Prevent Alzheimer's Disease, Potomac, MD, United States; b Campaign to Prevent Alzheimer's Disease, Potomac, Maryland, United States; c University of Las Vegas, Nevada, National Supercomputing Institute & Dedicated Research Network, Las Vegas, Nevada, United States; d Centre for AI, and Quantum Systems in Brain Research, CLARA, Prague, Czech Republic; e International Neurodegenerative Disorders Research Center, Prague, Czech Republic; f Editor-in-Chief, Journal of Aging Research & Lifestyle

## 1 Why brain performance is the new Frontier

With population trends moving toward an aging demographic and in an era of rapidly evolving healthcare that demands greater emphasis on prevention and personalization, prioritizing brain performance for the benefit of all has never been more critical. The complexities of brain functionality call for a strategic and multidisciplinary approach. Incorporating models from clinical science, translational medicine, geroscience, systems biology, engineering, social sciences, and even thermodynamics into the framework for brain performance offers a multidimensional view of how the brain functions and adapts under various conditions. These models provide new pathways to quantify, monitor, and optimize brain function, ensuring proactive attention to maintaining its resilience and efficiency [1,2].

### 1.1 So, what is brain performance?

Brain performance refers to the brain's capacity to maintain and optimize its cognitive, emotional, and functional abilities across a range of conditions. It is not simply about the absence of disease but how well the brain adapts, manages stress, and maintains functionality through dynamic and interconnected systems. This concept aligns with and expands upon the WHO's work on **vitality capacity**, emphasizing the importance of **intrinsic capacity**—the sum of all physical and mental capacities available to an individual across the lifespan [1,3]. The WHO's focus on **vitality** highlights the interaction of multiple physiological systems, including energy metabolism, neuromuscular function, and immune and stress responses that directly influence brain performance.

Brain performance can be seen as the **new frontier** in healthcare because it seeks to move beyond traditional diagnostic categories toward a more **integrated, multidimensional** understanding of how the brain functions in diverse conditions[4]. This approach focuses on early detection, ongoing monitoring, and proactive interventions to optimize brain function, such as monitoring vitality capacity to prevent frailty. As populations age and healthcare advances demand more personalized and preventive approaches, brain performance becomes critical not only for maintaining cognitive health but also for overall well-being.

A **Brain Watch** is an innovative concept aimed at crafting personalized, accurate, high-quality plans tailored to sustain brain performance and wellness [5]. By integrating insights from the study of frailty—such as the importance of exercise, nutrition, and comprehensive assessments—we can develop robust strategies that enhance brain adaptability, resilience, and robustness [6,7].

This perspective editorial will delve into the foundational need to develop personalized medicine techniques and advanced data analytics and promote cutting-edge methodology to monitor and optimize brain performance. With proactive care, regular monitoring, and personalized interventions, a **Brain Watch** will empower individuals and their health providers to navigate the complexities of aging while maintaining cognitive, functional, and behavioral excellence critical to overall total person performance.

This journey towards optimal sustained personal brain performance embraces an integrated approach, recognizing that the brain's vitality is a function of its integrity and reflects the body's overall efficiency.

## 2 Recasting brain health as brain performance

The concept of **brain performance** offers a dynamic and functional perspective that redefines how we approach Alzheimer's disease and other disorders that impair memory, movement, and mood. Traditional models often center around the notion of brain health as a binary outcome—either "unaffected" or "diseased." However, focusing on **performance** emphasizes the brain's capacity to function across a spectrum, accounting for resilience, adaptability, and even compensatory mechanisms in the face of pathology. This approach shifts attention from static measurements of brain deterioration to **how well the brain operates** under real-world conditions, such as managing cognitive load, regulating emotion, and maintaining motor control. By framing Alzheimer's disease and other chronic diseases of brain aging through the lens of performance, we can better understand why individuals with similar levels of pathology may exhibit vastly different functional abilities. Ultimately, recasting brain health as brain performance broadens our understanding of neurodegenerative disorders and encourages strategies to optimize brain function across the lifespan rather than merely managing decline.

Traditional medical models tend to focus primarily on the **pathological markers of decline**, such as the accumulation of amyloid plaques or neurofibrillary tangles in Alzheimer's disease. These models often portray brain health as a downward trajectory tied to the presence and progression of disease. In contrast, a **performance-based approach** broadens the scope to consider how well the brain continues to function despite these underlying pathologies. By emphasizing factors like **resilience**—the brain's ability to recover from stressors—**adaptability**, or how the brain adjusts to changing conditions and **reserve**, the extra capacity the brain can draw on to compensate for damage, we gain a more nuanced understanding of brain function. This shift recognizes that cognitive and functional capacity may be maintained or optimized, even in the presence of neurodegenerative changes.

This approach has significant implications for **clinical care**. Conversations between clinicians and patients may evolve from primarily focused on diagnosing and tracking pathology to **optimizing brain performance**. Clinicians can guide patients to focus on **building cognitive and functional reserves** through lifestyle interventions such as cognitive training, physical exercise, and managing comorbidities like hypertension or diabetes, which can impact brain function. By shifting the conversation towards **preserving and enhancing performance**, patients are empowered to take proactive steps to maintain independence and quality of life. This framework also supports the development of personalized care strategies, where interventions aim to slow disease progression and **enhance cognitive, behavioral, and functional performance** through strategies that leverage the body's capacity for resilience and adaptability. The shift from a pathology-centered model to one focused on **whole-body performance** encourages clinicians to adopt a more integrative dialogue with their patients, focusing on what they can do to optimize their brain function over time [8,9].

While the shift to a **performance-based model** and the development of personalized care strategies represents a compelling vision for the future of clinical care, these ideas are still **aspirational** in many ways. The practical implementation of such an approach will require significant advances in both research and clinical infrastructure, as well as medical training and education [10,11]. Nevertheless, these aspirations provide a crucial foundation for rethinking how we assess and enhance brain function and performance across a lifespan, moving beyond the limits (e.g., inability to obtain relevant longitudinal data of disease processes) of current pathology-driven models. To translate this vision into reality, we must develop approaches and technologies to monitor and measure brain performance accurately, precisely, and reliably. This brings us to the ***Brain Watch*** concept and the need to establish a framework for designing, building, and calibrating devices, methods, and means of communication that will enable us to track and optimize brain performance in real time.

## 3 Designing, building, and calibrating your "brain watch"

Having established the aspirational framework for understanding brain performance, the next step is to move towards **operationalizing** this vision: How will we design, build, and calibrate a "brain watch" as a chronometer for an individual's brain performance? This concept rests on three distinct yet interconnected facets: **designing, building**, and **calibrating** the “Brain Watch.” Each component shapes how we measure, understand, and improve brain function across various domains.

### 3.1 The design

The first step in this process is the **design phase**, where we develop an initial **matrix of constructs** that defines and characterizes brain performance. This matrix must be adaptable, allowing for future iterations and expansion as scientific knowledge and medical practices evolve. In essence, the design process will establish the foundational parameters that guide how we measure brain function, emphasizing **physiological performance** over traditional health concepts [12,13]. This shift highlights durability, functionality, and efficiency rather than simply the absence of disease [14].

The design process should focus on ten initial—leaving the possibility to include additional— constructs to define brain performance. Table 1 summarizes these constructs that provide a **multifaceted view of physiological performance** and offer the flexibility to evolve with scientific advancements.

**Table 1:** Brain performance constructs: these ten constructs provide the **blueprint** for designing a robust framework to monitor brain performance, setting the stage for the next phase: **building** the tools and technologies that will enable us to measure these constructs in real time. The adaptability of the matrix ensures that it will evolve with advancements in neurobiology, allowing for **calibration** and refinement as we move forward.

Construct	Argument	Example
**Homeostasis as Performance Efficiency**	Homeostasis can be reframed as the optimal performance of physiological systems in maintaining balance, especially under changing conditions.	The body's thermal performance ensures that core temperature remains within optimal limits during external stress.
**Vital Signs as Performance Metrics**	Vital signs serve as key performance indicators (KPIs) of the body's core functions. Blood pressure, heart rate, respiratory rate, and oxygen saturation reflect how efficiently these systems are operating.	Resting heart rate is a critical measure of cardiovascular performance, indicating how effectively the heart pumps blood and supplies oxygen.
**Laboratory Values as Performance Benchmarks**	Laboratory tests provide measurable benchmarks for biochemical and metabolic performance. For instance, blood glucose levels represent how well the body regulates energy, while liver enzymes indicate detoxification performance.	Glucose performance reflects the body's ability to regulate energy efficiently, with deviations suggesting issues in insulin pathways.
**Allostasis as Adaptive Performance**	Allostasis highlights how physiological systems adjust to changes to maintain stability.	During stress, the adrenal system's performance adapts by producing cortisol, which regulates energy and immune function.
**Cardiometabolic Performance**	Cardiometabolic health can be better understood as cardiometabolic performance, focusing on how the heart, lungs, and metabolic systems function under varying demands.	VO2 max is a powerful indicator of aerobic performance and overall cardiovascular and respiratory efficiency.
**Biomarkers of Aging as Performance Indicators**	Aging biomarkers, such as telomere length, can be viewed as indicators of aging performance, reflecting how aging impacts cellular and systemic functions.	Telomere performance signals how efficiently cells replicate and repair over time.
**Functional Performance**	Functional status can be reframed as functional performance, focusing on how well individuals perform everyday tasks and maintain independence.	Mobility performance measures how effectively a person can move and perform physical tasks, vital for quality of life.
**Body Composition as Performance Capacity**	Body composition, particularly the balance between muscle mass and fat, reflects physical performance capacity, indicating how well the body can perform physical activities.	Lean muscle performance is crucial for maintaining physical endurance and resilience against fatigue.
**Endocrine Performance**	Endocrine system performance emphasizes the regulation of hormones like insulin, cortisol, and thyroid hormones, which play crucial roles in metabolism, growth, and stress response.	Insulin performance is a critical measure of metabolic control, ensuring stable blood glucose levels.
**Immunological Performance**	The immune system's ability to fight infections can be assessed in terms of immunological performance, focusing on how efficiently it identifies and eliminates pathogens.	Immunological performance is gauged by the body's ability to mount a swift and controlled response to infections.

## 4 Cognition, behavior, and function as performance categories

A comprehensive approach to creating a ***Brain Watch*** must assess physiological and cognitive, behavioral, and functional performance. These dimensions are essential for understanding how individuals interact with their environment, process information, and maintain independence. Alzheimer's disease and other neurodegenerative disorders affect all these areas, making the ability to measure performance in each domain critical for early intervention and the development of personalized care strategies.

**Cognitive Performance** reflects how effectively the brain processes information, solves problems and engages in tasks involving memory, attention, and executive function. A performance-based approach shifts the focus from mere cognitive decline to evaluating how well individuals function cognitively under varying conditions. For example, cognitive performance can be assessed by examining how well individuals recall information, solve complex problems, or maintain focus under challenging circumstances. This shift to performance emphasizes the dynamic aspects of cognition rather than simply documenting decline.

**Behavioral Performance** involves emotional regulation, social interactions, and responses to environmental stressors, all of which are often disrupted in neurodegenerative diseases. Behavioral symptoms, such as apathy, anxiety, or depression, are common in conditions like Alzheimer's disease, and assessing behavioral performance helps clinicians understand the broader impacts of the disease on daily life and mental health. For instance, behavioral performance can be evaluated by assessing emotional resilience and adaptability, such as an individual's ability to manage stress, engage in social interactions, or maintain emotional stability.

**Functional Performance** focuses on how well individuals carry out daily activities, from basic self-care to more complex tasks like managing finances or household responsibilities. Declines in cognitive and behavioral performance often manifest first in functional tasks, providing early indicators of brain health issues before more overt cognitive symptoms appear. Functional performance can be assessed by monitoring an individual's ability to perform instrumental activities of daily living (IADLs), such as cooking, shopping, or managing medications.

## 5 Recasting frailty and SOMMA constructs into performance measures

The 13 frailty indices typically assess the physical, psychological, and functional decline that leads to vulnerability in older adults. These indices emphasize deficit accumulation, which is useful for identifying those at risk of adverse health outcomes. On the other hand, the SOMMA constructs may involve a broader range of indicators, potentially blending cognitive, emotional, and functional assessments.

By translating these frailty measures into **performance-based metrics**, the focus shifts from simply identifying deficits to evaluating how well an individual performs across key domains like **mobility, cognitive agility**, and **emotional regulation**. This allows for early detection and intervention aimed at improving or maintaining brain function (Table 2).

**Table 2:** Recasting frailty indices and SOMMA constructs into performance-based metrics for early detection and intervention.

Frailty construct	Performance metric
Physical Frailty	Physical performance capacity (e.g., strength, endurance, flexibility) in dynamic environments
Nutritional Status	Metabolic performance (e.g., monitoring nutrient processing to support cognitive and physical tasks)
Cognitive Decline	Cognitive performance (e.g., problem-solving ability, memory retention, decision-making speed)
Emotional or Psychological Decline	Behavioral performance (e.g., resilience to stress, adaptability in social contexts)

## 6 Consideration of the WHO vitality framework

The World Health Organization's (WHO) vitality capacity model represents a paradigm shift from traditional deficit-based assessments, such as frailty indices, toward an approach that monitors the body's intrinsic capacity to perform key physiological functions. According to the WHO, vitality encompasses the sum of all physical and mental capacities that an individual can draw upon, with a particular emphasis on energy levels, neuromuscular function, and immune response. This model is dynamic and adaptive, aligning closely with performance-based metrics, as previously discussed (Table 3).

**Table 3:** Comparison of frailty indices and WHO vitality framework: similarities and differences.

Category	Description
**Similarities**	
Physiological Assessment	Both frailty indices and the WHO vitality framework emphasize the assessment of multiple physiological systems (e.g., cardiovascular, respiratory, musculoskeletal) contributing to overall capacity.
Resilience and Recovery	Both frameworks recognize the importance of resilience and the ability to recover from stressors, aligning with the performance-based concepts of brain resilience and reserve.
Multifaceted Aging	The SOMMA constructs and the WHO framework acknowledge that healthy aging is multifaceted, involving interdependent physical, cognitive, and emotional dimensions.
**Differences**	
Risk vs. Proactivity	Frailty indices focus on identifying individuals at risk of decline, while the WHO model adopts a proactive approach, monitoring and maintaining vitality over time through early interventions.
Vulnerability vs. Capacity	Frailty indices and the SOMMA constructs emphasize vulnerability and risk, whereas the WHO vitality framework focuses on preserving capacity and maintaining functional ability, rather than solely preventing frailty.

**Developing Brain Performance Measures from These Models:** Frailty indices and the WHO vitality framework offer valuable input for developing brain performance metrics. The WHO vitality framework, focusing on the interplay between multiple physiological systems and their cumulative impact on capacity, supports developing performance measures for brain function.

A performance-based model for brain health would be consistent with the WHO approach by integrating physiological and cognitive metrics to track the brain's functionality across various states. While frailty indices provide insights into the accumulation of specific deficits, a brain performance framework would extend beyond deficit tracking to evaluate brain systems' efficiency, resilience, and adaptability over time.

The WHO vitality capacity framework and frailty indices offer complementary perspectives instrumental in developing brain performance metrics. The WHO's emphasis on intrinsic capacity aligns closely with a performance-based approach, highlighting the importance of continuous monitoring and proactive interventions to preserve brain function. These frameworks provide a solid initial foundation for constructing a comprehensive ***Brain Watch*** that integrates cognitive, behavioral, and functional performance with traditional physiological metrics.

## 7 Call to action: joining the brain watch coalition for advancing brain performance monitoring

The shift from focusing on **health** to **performance** marks a significant change in assessing brain and physiological function. Rather than adopting a static view of bodily conditions, this new perspective emphasizes dynamic and functional metrics, reflecting how well systems operate in real-time. This approach highlights three key aspects of performance:

1. **Efficiency**: How well physiological systems perform their respective tasks.
2. **Adaptability**: The ability of these systems to adjust to stress and challenges.
3. **Output**: The measurable outcomes, such as vital signs and lab values, that demonstrate system-level performance.

To develop a robust framework for brain performance, we must look beyond traditional health models and incorporate insights from other disciplines. Drawing from engineering, social sciences, and life sciences provides valuable perspectives that can help establish a comprehensive framework for brain performance. Below, several models from diverse research and scientific areas offer other helpful analogies for this effort:

1. **Control Systems Theory (Engineering)**: Control systems maintain stability in dynamic environments through feedback loops. Applied to brain function, this model could measure how neurological systems adjust and maintain cognitive, emotional, and motor functions in response to stressors. For example, monitoring homeostatic balance (e.g., neurotransmitter levels, energy usage) would provide insight into brain resilience.
2. **Human Factors Engineering (Ergonomics)**: This model focuses on optimizing human interaction with systems by accounting for cognitive load and environmental demands. In the context of brain performance, it provides a framework for understanding how cognitive load, stress, and fatigue affect performance under different conditions. Performance benchmarks could be created by studying multitasking and decision-making abilities under varying levels of demand.
3. **Systems Biology (Life Sciences)**: Systems biology examines complex interactions within biological networks. Applied to the brain, it offers a way to study how different regions and systems (metabolic, cognitive, emotional) interact to support overall brain performance. By mapping brain activity across these networks, we can assess how dysfunction in one area affects the entire system.
4. **Social Ecological Model (Social Sciences)**: This model examines how multiple layers of influence—ranging from individual to societal—affect outcomes. Applied to brain performance, it acknowledges that cognitive and emotional function are shaped not only by individual factors but also by social and environmental interactions. Monitoring changes in social engagement or environmental conditions could provide insight into maintaining brain health.
5. **Resilience Engineering (Risk Management)**: Resilience engineering focuses on how systems withstand disruptions and continue to function. In terms of brain performance, this model could assess how the brain responds to acute stressors (e.g., emotional trauma or cognitive overload) and recovers function, offering metrics of cognitive resilience.
6. **Thermodynamics and Energy Flow Models (Physical Sciences)**: Thermodynamic principles can be used to assess how efficiently the brain uses its energy reserves, especially during high-demand tasks. Monitoring the brain's energy allocation during various cognitive states (e.g., focused work, multitasking) would provide insights into fatigue and cognitive load.
7. **Game Theory (Mathematics/Social Science)**: Game theory explores decision-making under conditions of competition and cooperation. Applied to brain performance, it can model how cognitive systems optimize decision-making under uncertainty. Metrics based on game theory could assess cognitive flexibility, strategic thinking, and problem-solving capacity.
8. **Cognitive Load Theory (Educational Psychology)**: This theory focuses on the limits of working memory and how performance declines when these limits are exceeded. We can gain insight into mental fatigue and cognitive capacity by developing metrics that assess how well the brain processes information under different cognitive loads.

By integrating these interdisciplinary models, we can transition from a static health-based framework to a dynamic, performance-based model of brain function. This approach emphasizes continuous monitoring of efficiency, adaptability, and outcomes, providing a more nuanced understanding of how the brain operates in real-world environments. Ultimately, such a framework could inform the development of tools, such as a comprehensive "Brain Watch," that track cognitive, behavioral, and functional performance alongside traditional physiological metrics.

## 8 Conclusion

In conclusion, as we shift from traditional health-based assessments to a dynamic, performance-focused model, the opportunity to redefine how we understand and monitor brain function has never been more critical. Integrating interdisciplinary models—from control systems and human factors engineering to systems biology and resilience frameworks—provides a robust foundation for crafting a comprehensive **Brain Watch**. This tool has the potential to revolutionize how we assess brain performance across cognitive, behavioral, and functional domains, allowing for proactive interventions and real-time monitoring of brain health.

Members of the academic research community, clinical medicine, and experts from life sciences and engineering disciplines are invited to join *JarLIFE* and the **Brain Watch Coalition** workgroup, "*Crafting and Building a Brain Watch*" (http://www.brainwatchcoalition.org). By uniting diverse perspectives and expertise, we can collaboratively design and calibrate a cutting-edge performance-based framework to enhance scientific understanding and directly impact clinical practice and public health. Your contributions will be crucial in shaping the future of brain health assessment, ensuring that this ambitious initiative is rooted in the best medical, scientific, and engineering principles.

## Declaration of generative AI and AI-assisted technologies in the writing process

While preparing this work, the author used Grammarly to check for grammatical errors, improve clarity and conciseness, ensure appropriate tone, and verify the originality of the content. After using this tool/service, the author(s) reviewed and edited the content as needed and is responsible for the publication's content.

## Acknowledgment of contributions for “Building a Brain Watch”

The following organizations and institutions have played a significant role in supporting the development of ideas, research, and initiatives discussed in the editorial “*Building a Brain Watch*” and the support and development of the Brain Watch Coalition.

Their contributions are acknowledged with gratitude:

Pharmaceutical and Biotech Companies:

• Eli Lilly: For advancing Alzheimer's research and pioneering biomarkers and treatments over decades.
• Eisai: For its groundbreaking work in dementia treatments, including the development of lecanemab.
• Lundbeck: For its innovative approaches to Alzheimer's research and therapeutic developments.
• Alzheon: For its contributions to biomarker research and clinical trials for Alzheimer's treatments.

Technology Companies:

• Microsoft: For leveraging AI in healthcare, particularly in brainwave analysis and diagnostic innovations.
• IBM: For its advancements in brain modeling and AI applications in neurodegenerative disease research.

U.S. Government Agencies:

• U.S. Department of Veterans Affairs (VA): For its efforts to increase veteran participation in Alzheimer's research.
• U.S. Department of Defense (DoD): For supporting transformative Alzheimer's research through programs like the AZRP.
• U.S. Department of Health and Human Services (HHS): For its broader role in public health initiatives related to neurodegenerative diseases.
• U.S. Department of Agriculture (USDA): For its support in health-related research initiatives.

Academic and Medical Institutions:

• The Medical University of South Carolina, University of New Mexico, Cleveland Clinic, INOVA Fairfax Hospital, West Virginia University Medical Center, University of Geneva and the University of Oslo: For their contributions to clinical research and innovation in neuroscience.

International Collaborations:

• International Neurodegenerative Disorders Research Center (INDRC): For advancing global efforts in understanding neurodegenerative diseases.
• EU Horizon Europe Funding: This work is part of the CLARA project, which has received funding from the European Union's Horizon Europe research and innovation program under Grant Agreement No. 101136607. The views and opinions expressed are solely those of the author and do not necessarily reflect those of the European Union or its agencies

Legal, Medical and Strategic Support:

• Shulman Rogers: For providing legal guidance and support for initiatives related to brain health technologies.
• Louis C. Tripoli, MD, Paulo Pinho, MD, Giovanni Frisoni, MD, Ira Haraldsen, MD, PhD

## Declaration of competing interest

I have the following financial or non-financial interests that may be considered as potential conflicts of interest:
